# Mechanisms of Suppression and Enhancement of Photocurrent/Conversion Efficiency in Dye-Sensitized Solar-Cells Using Carotenoid and Chlorophyll Derivatives as Sensitizers

**DOI:** 10.3390/molecules17022188

**Published:** 2012-02-22

**Authors:** Yasushi Koyama, Yoshinori Kakitani, Hiroyoshi Nagae

**Affiliations:** 1 Faculty of Science and Technology, Kwansei Gakuin University, 2-1 Gakuen, Sanda 669-1337, Japan; 2 Kobe City University of Foreign Studies, 9-1 Gakuen-Higashimachi, Nishi-ku, Kobe 651-2187, Japan

**Keywords:** dye-sensitized solar cells, carotenoid and chlorophyll derivatives, singlet-triplet annihilation

## Abstract

The mechanisms of suppression and enhancement of photocurrent/conversion efficiency (performance) in dye-sensitized solar cells, using carotenoid and chlorophyll derivatives as sensitizers, were compared systematically. The key factor to enhance the performance was found to be how to minimize interaction among the excited-state dye-sensitizer(s). In a set of retinoic-acid (RA) and carotenoic-acid (CA) sensitizers, having *n* conjugated double bonds, CA7 gave rise to the highest performance, which was reduced toward RA5 and CA13. The former was ascribed to the generation of triplet and the resultant singlet-triplet annihilation reaction, while the latter, to the intrinsic electron injection efficiency. In a set of shorter polyene sensitizers having different polarizabilities, the one with the highest polarizability (the highest trend of aggregate formation) exhibited the higher performance toward the lower dye concentration and the lower light intensity, contrary to our expectation. This is ascribed to a decrease in the singlet-triplet annihilation reaction. The performance of cosensitization, by a pair of pheophorbide sensitizers without and with the central metal, Mg or Zn, was enhanced by the light absorption (complementary rather than competitive), the transition-dipole moments (orthogonal rather than parallel) and by the pathways of electron injection (energetically independent rather than interactive).

## Nomenclature

Some key concepts for those readers who are non-specialized in physical chemistry:

RA and CAs: RA (retinoic acid) and CA (carotenoic acid) are just the traditional classification depending on the conjugation length. RA5 can be written as CA5, as well.

*I*–*V* curve: The correlation between *I* (photocurrent) and *V* (photovoltage) characterizes the performance of a solar cell. When *V* = 0, the photocurrent flux is called *J*_sc_ (short-circuit photocurrent flux), whereas when *J*_sc_ = 0, the photovoltage is called *V*_oc_ (open-circuit photovoltage). The area of a rectangle surrounded by the *I*–*V* curve reflects *η* (solar energy-to-electricity conversion efficiency).

SVD (singular-value decomposition) and global-fitting: A method of spectral analysis to reconstitute the observed time-resolved difference spectra as SADS (species-associated difference spectra) (generated species *positive*, lost species *negative*) multiplied by time-dependent changes in population, and take a sum of them at each delay time. Perfect fitting can be obtained only when the kinetic model (the time constants of the relevant components) is (are) completely correct. Continuous fluctuation (noise) can be removed during these analytical processes.

CBE (conduction-band-edge): The lowest edge of the conduction band of the semiconductor to which electron can be injected into from the excited state of a dye-sensitizer.

IPCE (incident photon-to-current conversion efficiency): The efficiency of conversion, at each wavelength, from the incident photon to electron (%).

HOMO (the highest-occupied molecular orbital) and LUMO (the lowest-unoccupied molecular orbital): In linear or circular conjugated chains, a staircase-type set of electronic levels is formed in the order … LUMO+1 > LUMO > HOMO > HOMO–1 … symmetric with a line just in-between LUMO and HOMO.

## 1. Conjugation-Length Dependence of Excited-State Dynamics Affecting Photocurrent/Conversion Efficiency in Retinoic-Acid and Carotenoic-Acid Sensitizers

**Figure 1:**
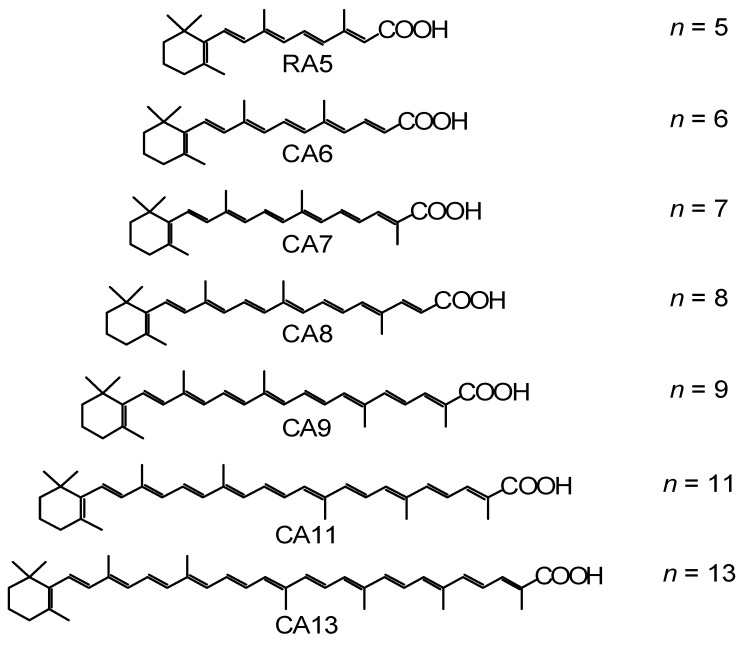
Chemical structures of retinoic acid (RA5) and carotenoic acids (CA6–CA13) having *n* = 5–13 conjugated double bonds.

Polyenes have a linear conjugated system, from which electrons can be injected into TiO_2_ when a carboxyl group is attached to facilitate binding and electron injection. As a set of sensitizers, we used a retinoic acid (RA) and carotenoic acids (CAs) having *n* = 5–13 double bonds (Figure 1). The dependence of their excited-state energetics and dynamics on the conjugation length (*n*) has been well-documented [1,2].

**Figure 2:**
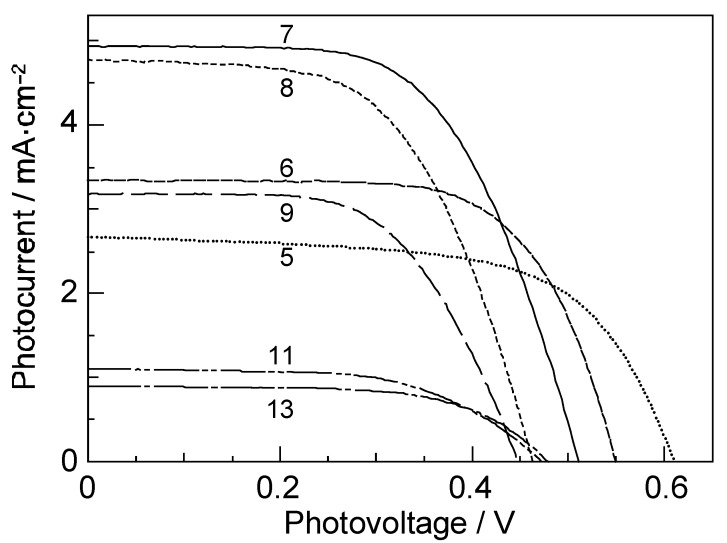
Conjugation-length (*n*) dependence of the *I*–*V* curves in solar cells using RA and CA sensitizers (reprinted from [3] with permission from Elsevier).

We first examined the conjugation-length dependence of the photocurrent and conversion efficiency (collectively called ‘performance’) of solar cells using the set of sensitizers, and tried to explain the results in terms of the excited-state dynamics of RA and CAs free in solution and bound to TiO_2_ nanoparticles in suspension. The highest performance was obtained with CA7; its decline toward CA13 was explained by the electron-injection efficiency, whereas that seen toward RA5 was explained partially in terms of triplet generation at later stages after excitation, as will be described below:

Figure 2 shows the *I–V* curves of solar cells using the set of sensitizers [3]. The short-circuit photocurrent density (*J*_sc_) is in the order, RA5 < CA6 < CA7 > CA8 > CA9 > CA11 > CA13, whereas the open-circuit photovoltage (*V*_oc_) is in the order, RA5 > CA6 > CA7 > CA8; CA8, CA9, CA11 and CA13 are exhibiting similar values.

**Figure 3:**
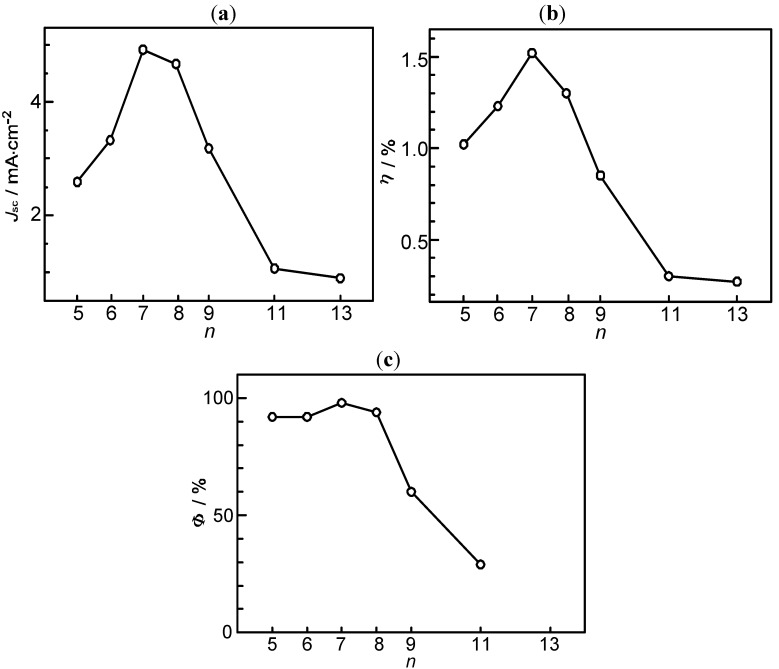
Conjugation-length (*n*) dependence of (**a**) the photocurrent (*J*_sc_) and (**b**) the conversion efficiency (*η*) in solar cells using the RA and CA sensitizers, and (**c**) the electron-injection efficiency (*Φ*) in the RA and CA sensitizers bound to TiO_2_ nanoparticles in suspension (reprinted from [4] with permission of the American Chemical Society).

Presumably, the coverage on the surface of TiO_2_ layer should be better-organized in the shorter-chain RA5, CA6 and CA7 sensitizers in the complete all-*trans* configuration; the longer-chain sensitizers tend to form *cis* isomers, as well. Open-circuit photovoltage (*V*_oc_) must reflect this situation. Figure 3a,b presents the conjugation-length dependence of short-circuit current density (*J*_sc_, hereafter simply called ‘photocurrent’) and the solar energy-to-electricity conversion efficiency (*η*, called ‘conversion efficiency’) [3]. 

The performance is at the maximum in CA7; they decline toward the shorter chain, in the order, CA6 and RA5, while toward the longer chain, in the order, CA8, CA9, CA11 and CA13, reflecting the trends of *J*_sc_ and *V*_oc_ mentioned above.

**Figure 4:**
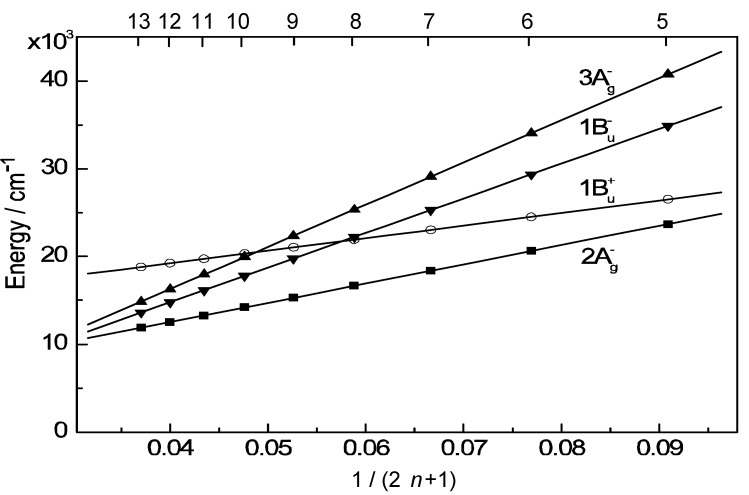
An energy diagram for the optically-allowed 1B_u_^+^ and optically-forbidden 2A_g_^–^, 1B_u_^–^ and 3A_g_^–^ states for RA an CAs having *n* = 5–13 conjugated double bonds (reprinted from [4] with permission of the American Chemical Society).

To understand the mechanisms giving rise to the above dependence of photocurrent and conversion efficiency on *n*, we examined the excited-state dynamics of the set of sensitizers: Figure 4 shows an energy diagram for the *π*-conjugated chains of RA and CAs with *n* = 5–13 [4]: the linear dependence of the optically-active 1B_u_^+^ state, as a function of 1 / (2*n* + 1), was determined by conventional electronic-absorption spectroscopy. 

The linear dependences of the optically-forbidden 1B_u_^–^, 3A_g_^–^ and 2A_g_^–^ states are transferred from those of bacterial Cars (*n* = 9–13) determined by the measurement of resonance-Raman excitation profiles [5]; the energies for CA8–RA5 were the extrapolation of these linear relations. According to the state ordering, after excitation to the 1B_u_^+^ state by the absorption of photon, (i) RA5, CA6, CA7 and CA8 are expected to internally convert, in the order, 1B_u_^+^ → 2A_g_^–^ → 1A_g_^–^ (the ground state), (ii) CA9 and CA10, in the order, 1B_u_^+^ → 1B_u_^–^ → 2A_g_^–^ → 1A_g_^–^ and (iii) CA11, in the order, 1B_u_^+^ → 3A_g_^–^ → 1B_u_^–^ → 2A_g_^–^ → 1A_g_^–^ as expected by the energy diagram.

On the basis of the above set of energy levels and internal conversion processes, we analyzed, by means of singular-value-decomposition (SVD) followed by global fitting, the time-resolved data matrices, for the set of RA5–CA11 sensitizers free in solution and bound to TiO_2_ nanoparticles in suspension.

**Figure 5:**
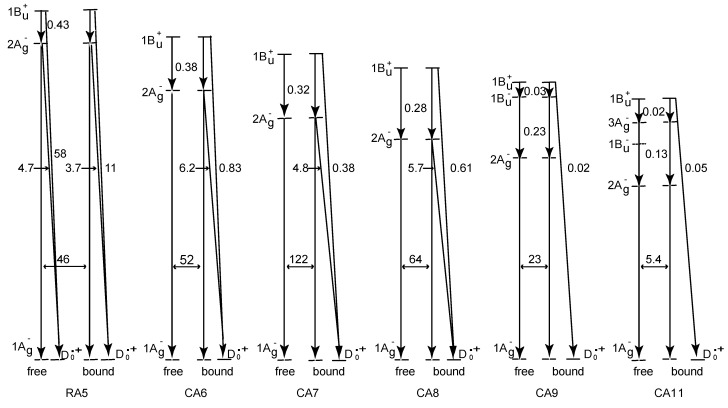
The pathways of internal conversion and electron injection for the RA and CA sensitizers free in solution and bound to TiO_2_ nanoparticles in suspension. The time constant for each pathway is shown in picoseconds (the T_1_ state generated together with the D_0_^•+^ state when bound which is not indicated) (reprinted from [4] with permission of the American Chemical Society).

Figure 5 presents the internal-conversion and electron-injection pathways and the relevant time constants for the free and bound states [4], which will be characterized below:

We start with the cases of RA and CAs free in solution: In RA 5, rapid transformation from the 1B_u_^+^ to the 2A_g_^−^ state followed by the generation of radical cation (D_0_^•+^) was observed. In CA6–CA8, rapid 1B_u_^+^ → 2A_g_^–^ transformation followed by the slow decay of the 2A_g_^–^ state was observed; here, no generation of D_0_^•+^ was seen. In CA9 and CA11, direct transformation from the 1B_u_^+^ to the 2A_g_^–^ state was not seen in the visible region, but rapid transformation from the 1B_u_^+^ to the 1B_u_^–^ state and that from the 1B_u_^+^ to the 3A_g_^–^ state, respectively, were seen in the near-infrared region. Their spectral patterns agreed with those of the 1B_u_^–^ and 3A_g_^–^ states of carotenoids, *i.e.*, neurosporene (*n* = 9) and lycopene (*n* = 11), respectively [6]. The time-dependent changes in population in CA9 showed extremely-rapid 1B_u_^+^ → 1B_u_^–^ transformation followed by the slower 1B_u_^–^ → 2A_g_^–^ transformation, whereas those in CA11, extremely-rapid 1B_u_^+^ → 3A_g_^–^ transformation followed by the slower 3A_g_^–^ → 2A_g_^–^ transformation (1B_u_^–^ was skipped). Both were followed by the 2A_g_^–^ → 1A_g_^–^ transformation.

Then, we proceed to the cases of RA5–CA11 bound to TiO_2_ nanoparticles in suspension: the singlet-excited states generated by the photo-excitation of the sensitizers bound to TiO_2_ were basically the same as those generated free in solution. The most conspicuous difference in the excited-state dynamics, in the bound state, is that the transient absorptions of the triplet (T_1_) and the radical-cation (D_0_^•+^) states appears immediately after electron injection (data not shown). The former transient absorptions agree, in energy, with those of the T_1_ states obtained by anthracene-sensitized photo-excitation, whereas the latter transient absorptions, with the stationary-state absorptions of radical cation obtained electrochemically, both free in solution. The generation of the apparent D_0_^•+^ + T_1_ state, however, drastically influences the dynamics of singlet-excited states (note that only the D_0_^•+^ state is shown in Figure 5). In RA5–CA8, the generation of the D_0_^•+^ + T_1_ state substantially accelerates the decay of both the 1B_u_^+^ and 2A_g_^–^ states, showing efficient electron injection from these excited states into TiO_2_. In CA9 and CA11, on the other hand, it accelerates the decay of *not* the 2A_g_^–^ state *but* the 1B_u_^+^ state, showing that electron injection was taking place only from the latter. This apparent D_0_^•+^ + T_1_ state stayed for 10 ps in RA5–CA8 and for 1.0 ps in CA9 and CA11, as far as the time range of pump-probe electronic-absorption spectroscopy.

Table 1 lists the electron-injection efficiencies through the 1B_u_^+^ and 2A_g_^–^ channels and a sum of the two, for the set of RA and CAs [7], which were calculated by the use of those time constants. The conjugation-length dependence of the total electron-injection efficiency (*Φ*) is depicted in Figure 3c. The highest efficiency was seen in CA7 (almost unity); the decline toward the longer-chain, *i.e.*, CA7 > CA8 > CA9 > CA11, reflects the intrinsic excited-state dynamics of the Car conjugated chain. However, the decline toward CA6 and RA5 is left unexplained. Table 2 shows that the values of one electron-oxidation potential systematically lowers with *n* [7], a trend which predicts the electron-injection efficiency monotonically increasing with *n*, all the way from *n* = 5 to 11, contrary to the observation in the fabricated solar cells.

**Table 1:** Electron-injection efficiencies through the 1B_u_^+^ and the 2A_g_^–^ channels and a sum of them, calculated by the use of time constants shown in Figure 5 (reprinted from [7] with permission of MDPI Publishing).

	RA5	CA6	CA7	CA8	CA9	CA11
**1B_u_^+^ channel**	0.04	0.31	0.46	0.31	0.60	0.29
**2A_g_^-^ channel**	0.88	0.61	0.52	0.63	–	–
**Sum**	0.92	0.92	0.98	0.94	0.60	0.29

**Table 2:** One-electron oxidation potentials in dichloromethane (in V) (reprinted from [7] with permission of MDPI Publishing).

	RA5	CA6	CA7	CA8	CA9	CA11
***E*** **_ox_ (** ***vs*** **. Ag/AgCl)**	1.08	0.97	0.87	0.80	0.77	0.71

We have observed the generation of ‘the D_0_^•+^ + T_1_ state’ just by transient absorptions, which does not decay at all in the time scales mentioned above. Therefore, we do not know, at this moment, what we now call ‘the D_0_^•+^ + T_1_ state’ is either ‘a *combined* D_0_^•+^ + T_1_ state’ or ‘a *mixture* of the D_0_^•+^ state and the T_1_ state’. We have applied submicrosecond pump-probe spectroscopy to examine the later stages after excitation. Therefore, we focused our attention on the later time region.

**Figure 6:**
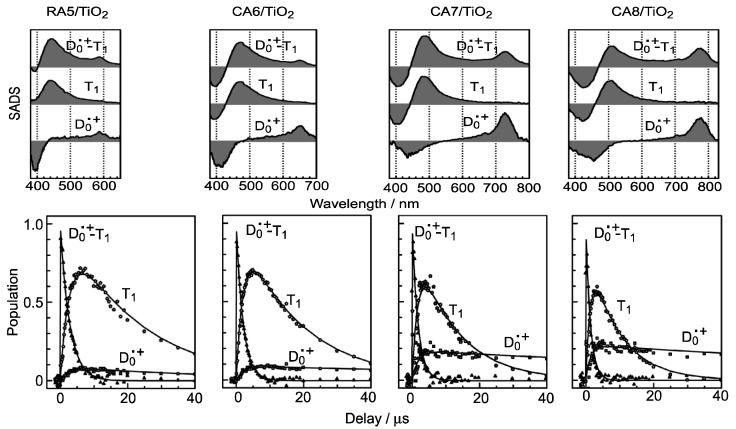
SADS (upper panels) and time-dependent changes in population (lower panels), obtained by singular-value decomposition (SVD) followed by global fitting, for RA5–CA8 bound to TiO_2_ nanoparticles in suspension (reprinted from [4] with permission of the American Chemical Society).

Figure 6 shows the results of the analysis of submicrosecond time-resolved data for the four shorter-chain RA and CAs [4]. Here, a relaxation mechanism, including the splitting of a combined D_0_^•+^ + T_1_ state into a pair of the D_0_^•+^ and T_1_ states, has been nicely explained. The first spectral patterns (upper panels) show that the T_1_/D_0_^•+^ population ratio in the *combined* D_0_^•+^ + T_1_ state increases toward RA5. Consistently, the time-dependent changes in population (lower panels) show that the ratio of the *split* T_1_/D_0_^•+^ species also increases toward RA5.

**Figure 7:**
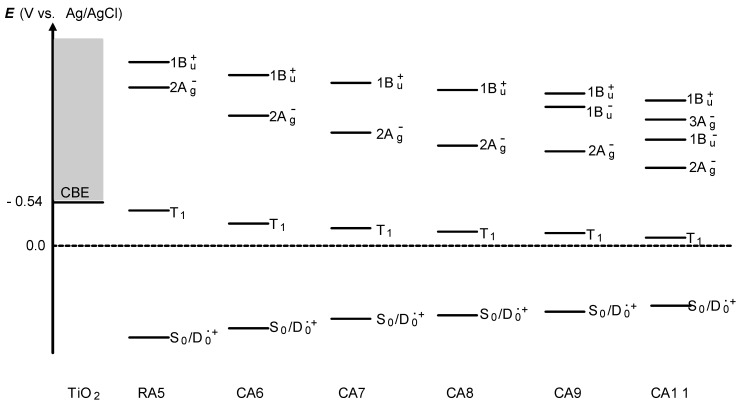
The energies of the singlet, triplet and redox states of RA5 and CA6–CA11 in comparison to that of the conduction-bond edge (CBE) of TiO_2_ (reprinted from [4] with permission of the American Chemical Society).

Table 3 lists the quantum yields for the D_0_^•+^ and T_1_ species (*Φ*_D_ and *Φ*_T_) calculated by the use of the relevant time constants in Figure 5 [4]. The efficiency of electron injection (*Φ*_D_) declines toward RA5. This trend solves the above-mentioned contradiction in the dependence on *n* shown in Figure 3, *i.e.*, (a) and (b) *vs.* (c). Complementally, the efficiency of triplet generation (*Φ*_T_) increases toward RA5, which enhances singlet-triplet annihilation to be described in Section 2.1. Finally, we will propose the mechanisms of charge-separation and charge-recombination, which generates the radical-cation and triplet species of RA and CAs on the surface of TiO_2_ nanoparticles. Figure 7 presents the energies of the singlet, triplet and redox states of RA5 and CA6–CA11, in reference to that of the conduction-band edge (CBE) of TiO_2_ [4]. Importantly, the energy gap between the CBE and the T_1_ levels decreases toward RA5, which explains the increasing order of the triplet generation mentioned above.

**Table 3:** The time constants of transformation from the D_0_^●^^+^−T_1_ complex to the D_0_^●^^+^ and T_1_ states (*k*_d_^−1^ and *k*_t_^−1^) and the D_0_^●^^+^ and T_1_ lifetimes (*k*_d0_^−1^ and *k*_t0_^−1^). The partition efficiencies from the D_0_^●^^+^−T_1_ complex to the D_0_^●^^+^ and T_1_ states (*Φ*_D_ and *Φ*_T_) are also listed (reprinted from [7] with permission of MDPI Publishing).

	RA5-TiO_2_	CA6-TiO_2_	CA7-TiO_2_	CA8-TiO_2_
***k*_d_^−1^ (** **μs)**	34	22	9.4	5.9
***k*_t_^−1^ (** **μs)**	3.1	2.7	2.1	2.0
***k*_t0_^−1^ (** **μs)**	22	18	12	9.0
***k*_d0_^−1^ (** **μs)**	~50	~150	~150	~150
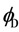 **(%)**	8	11	18	25
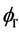 **(%)**	92	89	82	75

**Figure 8:**
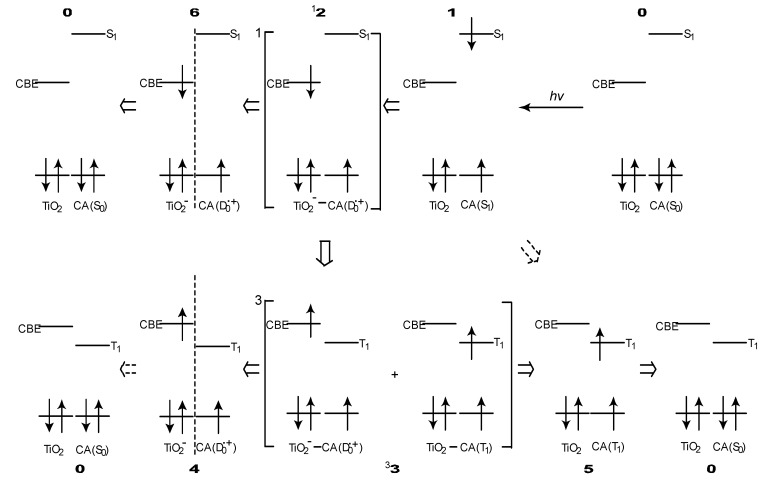
Excitation, electron transfer and relaxation dynamics in a typical RA or CA bound to TiO_2_ nanoparticles in suspension. Mechanisms of electron injection as well as charge recombination, following intersystem crossing and exciplex formation, to generate triplet (T_1_) and radical cation (D_0_^●+^) species of the sensitizer. Each numbered state is expressed by a combination of TiO_2_ and CA in the ground, redox or excited states (reprinted from [4] with permission of the American Chemical Society).

Figure 8 proposes the excited-state dynamics in a typical CA that is bound to TiO_2_ [4]: (i) Process **0** → **1**: Upon absorption of photon, electron is transferred to a higher singlet level (S_1_). (ii) Process **1** → ^1^**2**: Electron injection takes place to generate a charge-separated state having a singlet character on the CA–TiO_2_ boundary. (iii) ^1^**2** → **6**: the electron is transferred further into TiO_2_ to form a stable charge-separated state. (iv) **6** → **0**: the reverse electron transfer followed by charge recombination takes place to relax into the ground state. This is a series of changes among the singlet-excited and redox states having *a singlet character*.

Now, we will consider the generation of the triplet-excited and radical-cation states both having *a triplet character* in the excited state: (v) Process ^1^**2** → ^3^**3**: When there is a strong spin-orbit coupling in the charge-separated state having the singlet character, it can transform, by the inversion of spin, into the charge-separated state having a triplet character. When the energy gap between the CBE and the T_1_ levels is small, the resultant charge-separated state can transform further into *a charge-transfer complex* (^3^**3**) consisting of the charge separated (TiO_2_^–^–CA(D_0_^•+^)) state and the neutral (TiO_2_–CA(T_1_)) state. This is exactly what we called ‘the *combined* D_0_^•+^ + T_1_ state’ (*vide supra*), because the former component gives rise to the radical-cation electronic absorption, whereas the latter component, the T_1_-state electronic absorption of CA.

In ^3^**3**, the relative contribution of the T_1_-state CA becomes larger when the energy gap between the CBE of TiO_2_ and the T_1_ states of CA becomes smaller (see Figure 7); this is actually evidenced by the spectral pattern of the D_0_^•+^ + T_1_ state (see Figure 6). This charge-transfer complex can split into two independent components as follows: (vi) ^3^**3** → **4**: It transforms into the pure D_0_^•+^ state of CA, the lifetime of which can be very long when the electron is trapped far from the surface of TiO_2_ particles in suspension. (vii) ^3^**3 **→ **5**: it can transform into the T_1_ state of CA, which decays with an intrinsic T_1_ lifetime.

Most importantly, the T_1_/ D_0_^•+^ ratio in the charge-separated state ^3^**3** and split states (**4** and **5**) is determined by the gap between the CBE of TiO_2_ and the T_1_ state of RA and CAs. In summary, the mechanisms of electron injection immediately after excitation to the 1B_u_^+^ (S_1_) state and the charge recombination of the TiO_2_^–^–Car (D_0_^•+^) pair to form triplet Car, after the intersystem crossing and the formation of charge-transfer complex, have been revealed by the analysis of the ps and μs time-resolved data obtained by pump-probe spectroscopy of RA and CAs bound to TiO_2_ nanoparticles in suspension. The conjugation-length (*n*) dependence of the initial excited-state dynamics has nicely explained the photocurrent and conversion efficiency of solar cells using the RA and CA sensitizers, *i.e.*, the maximum at *n* = 5 and the decline toward *n* = 11. On the other hand, the decline toward *n* = 5 has been explained in terms of the generation of radical cation at later stages. Another possibility of ‘singlet-triplet annihilation’ using the T_1_ state generated will be examined in the following Section 2.1.

## 2. Singlet-Triplet Annihilation Mechanism; Quenching Singlet Excitation of RA and CAs

### 2.1. Dependence of Photocurrent/Conversion Efficiency on the Dye Concentration in CA7-Sensitized Solar Cells

**Figure 9:**
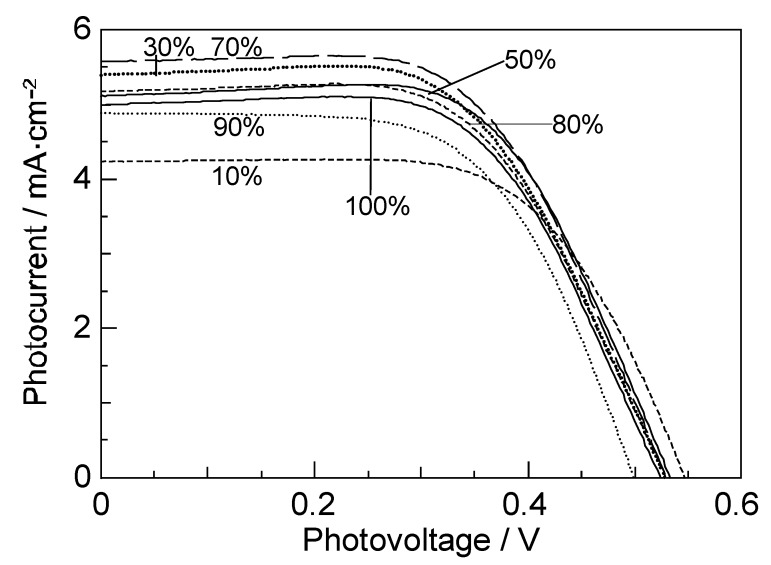
Concentration dependence of the *I*–*V* curves in CA7-sensitized solar cell (reprinted from [3] with permission of Elsevier).

**Figure 10:**
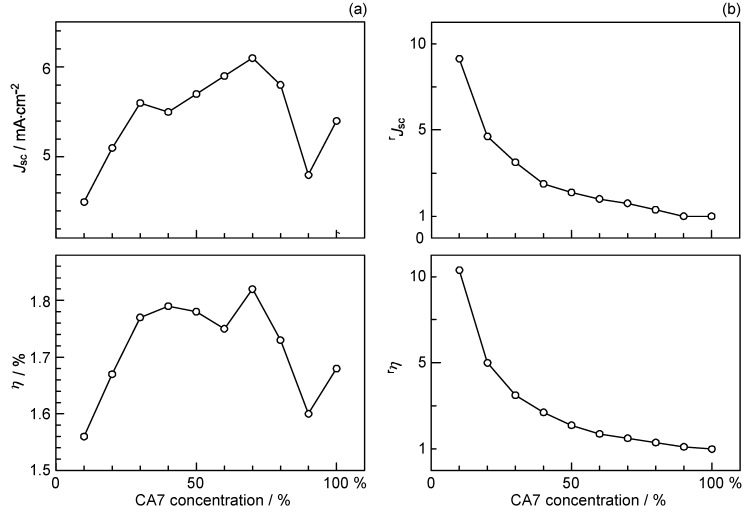
Effects of dilution with a spacer (deoxycholic acid) on (**a**) the photocurrent (*J*_sc_) and conversion efficiency (*η*) and (**b**) the relative photocurrent (^r^*J*_sc_) and conversion efficiency (^r^*η*) of CA7-sensitized solar cells. To obtain ^r^*J*_sc_(X) at a mole fraction X, for example, *J*_sc_(X) was scaled against concentration, and, then, a ratio was taken in reference to the value with no dilution. Thus, ^r^*J*_sc_(X) = *J*_sc_(X)/X/*J*_sc_(X = 1) and ^r^*η*(X) = *η*(X)/X/*η* (X = 1) (reprinted from [3] with permission of Elsevier).

Figure 9 shows a set of *I*–*V* curves of CA7-sensitized solar cells, when the sensitizer was diluted with a spacer, deoxycholic acid. Figure 10a shows the concentration dependence of *J*_sc_ and *η* [3]. Both parameters exhibit similar and unique concentration dependence, which can be characterized as follows: (i) At 100%, these values are medium among all the values at the different concentrations. (ii) On going from 100% to 90%, the values exhibit a sudden drop. (iii) Then, they increase up to a maximum at 70%. (iv) From 70% down to 30%, the values gradually decrease. (v) Below 30%, they decrease steeply toward the values at 10%.

**Figure 11:**
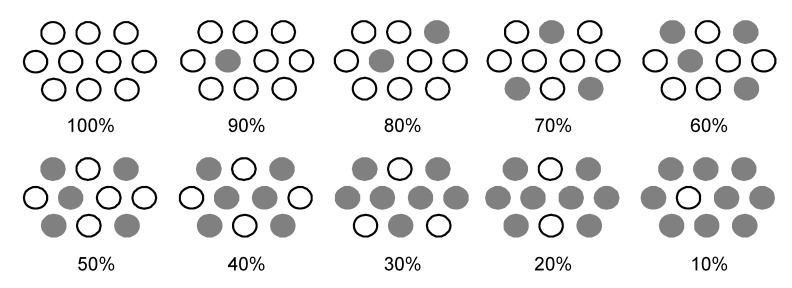
Typical arrangements of the dye (○) and spacer (●) molecules on the TiO_2_ surface formed during the processes of dilution of the former with the latter (reprinted from [3] with permission of Elsevier).

We propose four different forms of excitation based on the results shown in Figure 11 [3], where the dye molecules (○) are diluted with the spacer molecules (●): (i) at 100%, a coherent excitonic excitation takes place in an aggregate of dye molecules (we call this ‘coherent delocalized excitation’). (ii) At 90%, this excitation is destroyed by a spacer molecule that functions as a defect. (iii) At 70%, a localized excitation on a single molecule can migrate from one to another in different directions. This ‘migrating excitation’ must become most efficient when the dye concentration becomes around ^2^/_3_, because branched routes for the migrating excitation are formed. (iv) At 30%, the dye molecules become isolated being intervened by a larger number of spacer molecules. This ‘isolated excitation’ must become the largest in number when the dye concentration becomes around ^1^/_3_.

Based on the above three different types of singlet excitation on the TiO_2_ layer and the generation of the triplet state, and the resultant singlet-triplet annihilation as an intrinsic property of CAs bound to TiO_2_, we propose a possible mechanism to explain the unique concentration dependence of photocurrent/conversion efficiency in the fabricated CA7-sensitized solar cell (see Figure 10a and Figure 11): (i) In a coherent delocalized excitation at 100%, there is a good chance that the widely-expanded singlet excitation reaches a dye molecule in the T_1_ state to cause singlet-triplet annihilation. (ii) In a partially-destroyed delocalized excitation at 90%, the advantage of the widely-expanded coherent excitation in electron injection is lost, but there is still a chance of collision between ‘a partially-expanded delocalized singlet excitation’ and a localized triplet excitation to annihilate the former. (iii) In the localized excitation migrating along one of the branched routes at ~70%, there is a much less chance of collision with a triplet excitation, unless it is located on the particular route. (iv) In an isolated singlet excitation, there is no chance of its collision with an isolated triplet excitation. Then, the photocurrent/conversion efficiency decreases linearly with the decreasing number of the localized excited-dye molecules.

The relative photocurrent (^r^*J*_sc_) and conversion efficiency (^r^*η*) are depicted in Figure 10b (see the caption for their definition). Their concentration dependence indicates that changes in the singlet excitation of the dye molecules take place continuously, and the relative performance (^r^*J*_sc_ and ^r^*η*) becomes systematically enhanced until 9–10 times on going from the first to the last form of singlet excitation.

To summarize, the dependence of the photocurrent and conversion efficiency of the CA7-sensitizerd solar cell, on the dye concentration, has been explained in terms of changes in the form of singlet excitation of the sensitizer molecules on the surface of TiO_2_ layer, *i.e.*, coherent delocalized excitation → localized migrating excitation → isolated excitation. There is a good chance of substantial enhancement in performance, if we succeeded in achieving only the localized excitation, keeping the total number of excited-state dye molecules the same.

The substantially reduced performance at the 100% dye concentration is ascribable to singlet-triplet annihilation reaction. Therefore, the decrease in the photocurrent/conversion efficiency of solar cells from the CA7 sensitizer toward the RA5 sensitizer (see Figures 3a,b) can now be explained also by the effect of singlet-triplet annihilation among the sensitizer molecules on the surface of the TiO_2_ layer, in addition to the effect of the increasing triplet generation described in Section 1.

### 2.2. Suppression of Photocurrent/Conversion Efficiency in Polyene Sensitizers Having Higher Polarizability (the Higher Trend of Aggregate Formation)

**Figure 12:**
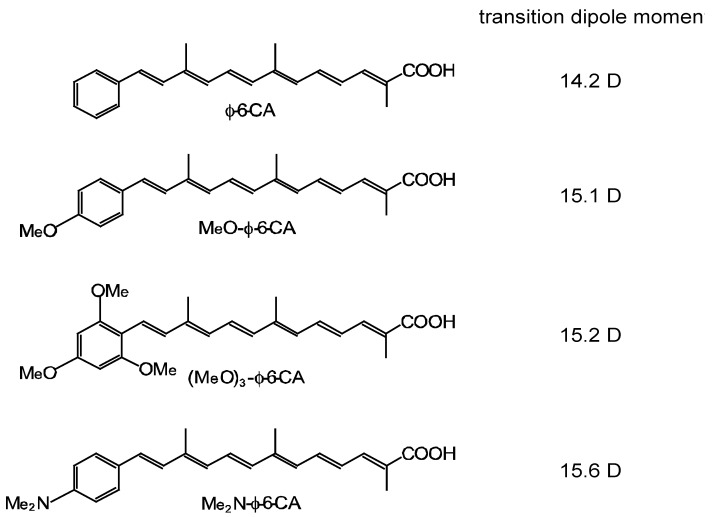
Chemical structures of a set of four polyene sensitizers with the increasing polarizability and, as a result, the increasing tendency of aggregate formation (reprinted from [8] with permission of Elsevier).

We prepared a set of four sensitizers having different polarizabilities and, as a result, different tendency of aggregated formation, and examined changes in the photocurrent/conversion efficiency of fabricated solar cells, depending on the dye concentration and the light intensity. The most-aggregate-forming dye exhibited the enhancement of performance by lowering the dye concentration and the light intensity, supporting the idea of singlet-triplet annihilation:

Figure 12 shows the structures of four different polyene sensitizers that were used for fabricating the solar cells [8]. The common skeleton of the sensitizers is the benzene ring connected to a short polyene (*n* = 6), to the end of which an electron-withdrawing carboxyl group is attached (*Φ*-6-CA); to the opposite end of the benzene ring the MeO-, (MeO)_3_- or Me_2_N- electron-donating group is attached to realize systematically the electron push-pull system in the latter set of sensitizers.

The set of polyene sensitizers are named *Φ*-6-CA, MeO-*Φ*-6-CA, (MeO)_3_-*Φ*-6-CA and Me_2_N-*Φ*-6-CA; the polarizability of polyene to enhance van der Waals intermolecular interaction to form aggregates is supposed to increase in this order. Actually, the transition-dipole moment calculated by the use of the molar extinction coefficient (*ε*) was in the order, 14.2, 15.1, 15.2 and 15.6 D, and the tendency of aggregate formation, judged by the blue-shift of the 1B_u_^+^ absorption band, was seen in the same order (data not shown).

**Figure 13:**
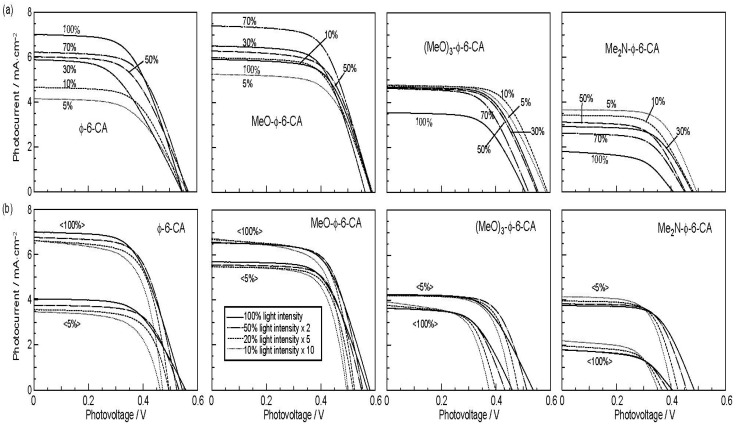
(**a**) The concentration dependence and (**b**) the light-intensity dependence (at two different concentrations) of the *I–V* curves in solar cells using the four sensitizers having different polarizabilities (see Figure 12) (reprinted from [8] with permission of Elsevier).

Figure 13a shows the concentration dependence of the *I*–*V* curves of solar cells using the above set of sensitizers. In the least-polarizable sensitizer, *Φ*-6-CA, the photocurrent (*J*_sc_) was the highest at 100% and monotonously decreased toward the lower concentration. 

In the most-polarizable sensitizer, Me_2_N-*Φ*-6-CA, on the other hand, the photocurrent was the lowest at 100% and monotonously increased toward the lower concentration. The latter change is contrary to our expectation, and can be explained only in terms of singlet-triplet annihilation. At 100%, the delocalized excitonic excitation should be generated due to the aggregate formation, which can be readily annihilated by collision with the triplet species within the expanded, excitonically-excited region. The chance of this singlet-triplet annihilation must become smaller by lowering the dye concentration.

Figure 13b shows the dependence of the *I*–*V* curves of the solar cells on the light intensity at two different dye concentrations (5% and 100%) [8]. In the least-polarizable sensitizer, *Φ*-6-CA, the photocurrent decreased with the lowering light intensity. On the other hand, in the most-polarizable sensitizer, Me_2_N-*Φ*-6-CA, the photocurrent increased, instead, with the lowering light intensity. The latter change is contrary to our expectation, and can be explained only in terms of singlet-triplet annihilation, because the generation of both the singlet and triplet excitation must become suppressed at the lower light intensity.

**Figure 14:**
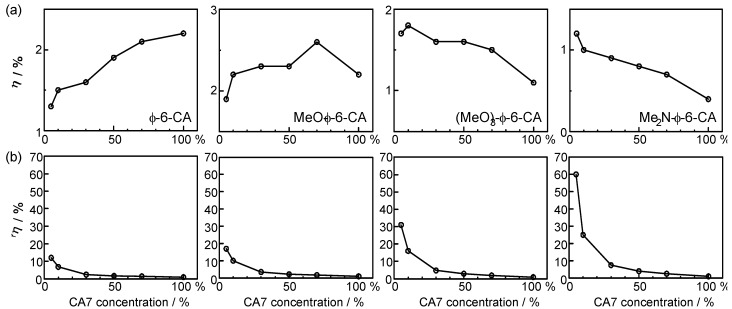
Concentration dependence of (**a**) the conversion efficiency (*η*) and (**b**) the relative conversion efficiency (^r^*η*) in solar cells using the four sensitizers with increasing polarizabilities (reprinted from [8] with permission of Elsevier).

Figure 14a plots the concentration dependence of conversion efficiency (*η*) for the set of polyene sensitizers [8]. In the least-polarizable sensitizer, *Φ*-6-CA, the conversion efficiency monotonously decreased, while in the most-polarizable sensitizer, Me_2_N-*Φ*-6-CA, it monotonously increased with the lowering dye concentration. In the second-least polarizable sensitizer, MeO-*Φ*-6-CA, conversion efficiency exhibits a maximum at 70%, while in the second-most polarizable sensitizer, (MeO)_3_-*Φ*-6-CA, it exhibits a maximum at 5%. Figure 14b shows that ^r^*η* increased in the order, *Φ*-6-CA < MeO-*Φ*-6-CA < (MeO)_3_-*Φ*-6-CA < Me_2_N-*Φ*-6-CA; it is ~60 times in the last sensitizer [7].

To summarize, the absence or presence of singlet-triplet annihilation has been demonstrated by lowering the dye concentration and the light intensity in solar cells by the use of the four sensitizers having the increasing polarizability and, as a result, the increasing tendency of aggregate formation. The least polarizable (the least aggregate-forming) sensitizer gave rise to the decreasing conversion efficiency with the decreasing dye concentration and light intensity, whereas the most polarizable (the most aggregate-forming) sensitizer gave rise to the increasing conversion efficiency with the decreasing dye concentration and light intensity. The four different patterns, in the dependence on the dye concentration and the light intensity, can be used as a standard to examine the degree of aggregate formation and the absence or presence of singlet-triplet annihilation in a new sensitizer.

## 3. Enhancement of Photocurrent/Conversion Efficiency by Preventing Singlet-Triplet Annihilation in Car–Phe Adduct and Chl *c*_2_ Sensitizers

### 3.1. Pheophorbide–Car Adduct: Energy Transfer and Electron Transfer from the Car to Phe Moiety

While searching for a sensitizer of Chl *a* derivative having a cyclic conjugated system, we found that pheophorbide *a* (Phe *a*) having the chlorin skeleton gave rise to reasonably-high photocurrent and conversion efficiency. Electron transfer from a neutral Car to Phe *a* radical cation (Phe *a*^●+^) can prevent the charge recombination in the TiO_2_^–^∙∙∙Car^●+^ charge-separated state, in the order, TiO_2_^–^∙∙∙Phe^+^–Car → TiO_2_^–^∙∙∙Phe–Car^●+^. Actually, the Car spacers enhanced the photocurrent and conversion efficiency of a solar cell using a Phe sensitizer.

**Figure 15:**
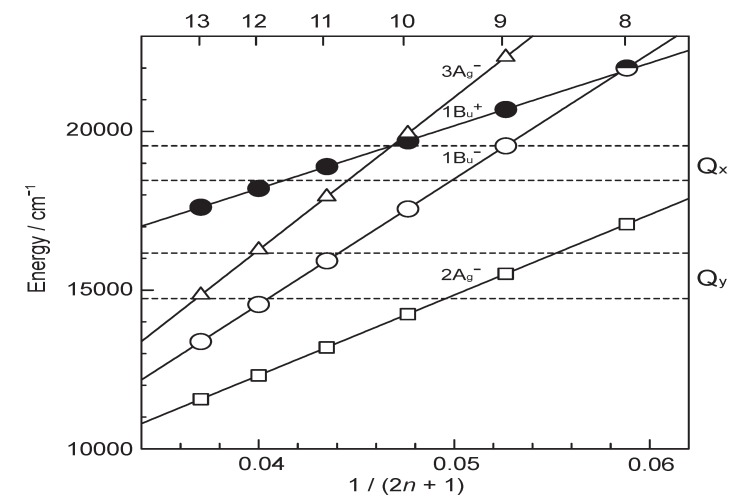
The energies of the optically-allowed 1B_u_^+^ and the optically-forbidden 2A_g_^–^, 1B_u_^–^ and 3A_g_^–^ states of Cars and those of the Q_x_ and Q_y_ states of Phe *a* (Phe *y*). Shorter-chain Cars (*n* = 8 and 9) have a better chance of singlet-energy transfer from Car to Phe *a* (1B_u_^+^ → Q*_x_*) (reprinted from [7] with permission of MDPI Publishing).

We found no signs of singlet-energy transfer in the above experiments, even by the use of the shortest-chain Cars (*n* = 8 and 9) having the higher singlet energies (1B_u_^+^) than those of Phe *a* (Q*_x_*) (see Figure 15 [7]). 

**Figure 16:**
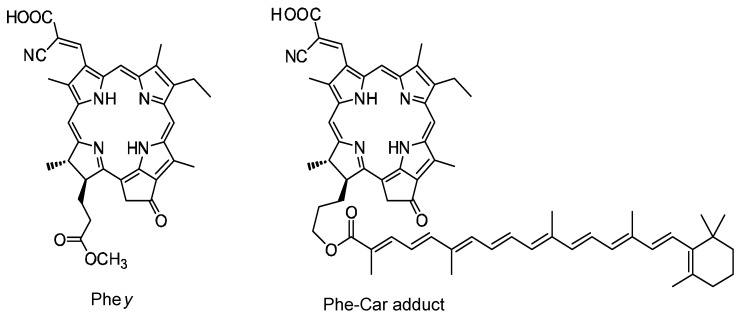
Chemical structures of Phe *y* and Phe–Car adduct (reprinted from [9] with permission of Elsevier).

We suspected that the direct van der Waals contact and the correct orientations of the transition dipoles between the Car and the Phe *a* moieties may be necessary to facilitate efficient singlet-energy transfer. Then, we synthesized an adduct sensitizer consisting of Phe *y* (modified from Phe *a*) and Car, which *actually* realized the singlet-energy transfer from the Car to the Phe moiety (see Figure 16), in addition to electron transfer, enhancing photocurrent/conversion efficiency. Further, the Car moiety, connected by single bonds to the Phe *y* moiety, could prevent the aggregate formation and the resultant singlet-triplet annihilation, which was evidenced by the suppression of performance by lowering the light intensity.

Figure 16 presents the chemical structures of ‘Phe *y*’ sensitizer and ‘Phe–Car adduct’ [9]. Phe *y* has a structure similar to Phe *a*, in which the carboxyl group attached to ring A is replaced by the ethenyl-cyano-carboxyl group that was supposed to enhance electron injection. Phe–Car adduct consists of the Phe *y* and *β*-apo-8’-carotenoyl (*n* = 9) moieties, and, therefore, singlet energy-transfer becomes possible from the latter to the former.

In fact, the *π*-conjugated systems of the two moieties are connected loosely through a couple of single bonds so that their electron clouds can overlap with each other to facilitate efficient electron transfer and, in addition, the 1B_u_^+^ transition moment of the Car moiety and the Q*_x_* transition moment of the Phe moiety can be set parallel to facilitate the 1B_u_^+^ to Q*_x_* singlet-energy transfer. (Note that the Q*_x_* transition moment of Phe and the 1B_u_^+^ transition moment of Car should be actually overlapped with each other; the figure is just for simplification.) When the adduct is bound to the TiO_2_ surface, the intervening bulky Car group may prevent the formation of Phe *y* aggregate and, as a result, suppress the singlet-triplet annihilation reaction.

**Figure 17:**
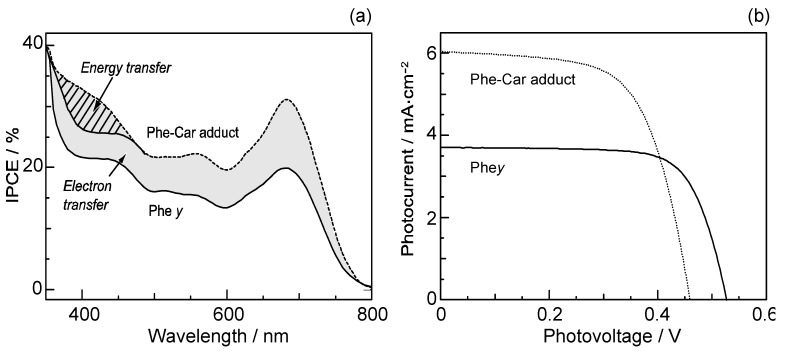
(**a**) The IPCE profiles and (**b**) the *I–V* curves of solar cells sensitized by Phe *y* and Phe–Car adduct (reprinted from [9] with permission of Elsevier).

Figure 17a compares the IPCE profiles of the solar cells using the Phe *y* and Phe–Car adduct sensitizers [9]. In the longer-wavelength region (500–800 nm), we see the shift of basically the same IPCE profile from the former to the latter. The shift of the IPCE profile in this region is ascribable to *electron transfer* from the Car to the Phe *y* moiety. In the shorter-wavelength region (370–470 nm), a bump is observed in the IPCE profile of Phe–Car adduct. Definitely, this is ascribable to *singlet-energy transfer* from the Car to the Phe *a* moiety. Figure 17b compares the *I*–*V* curves for the two sensitizers [9]: the Phe *y* sensitizer gives rise to a higher *V*_oc_ value, while the adduct sensitizer, a higher *J*_sc_ value. The former observation presumably reflects the better packing of the Phe *y* sensitizers on the TiO_2_ surface, because the bulky Car moiety in Phe–Car adduct must prevent ordered surface coverage. The latter observation must reflect the larger photocurrent due to the electron transfer and energy transfer from the Car to the Phe moiety as mentioned above. The introduction of the Car moiety enhances *J*_sc_ by 1.6 times and *η* by 1.3 times. The *E*_ox_ values of Phe–Car adduct reflected those of the Car moiety (0.95 V) and the Phe *y* moiety (1.17 V), which supports the idea of electron transfer from both the Car and the Phe moiety.

**Figure 18:**
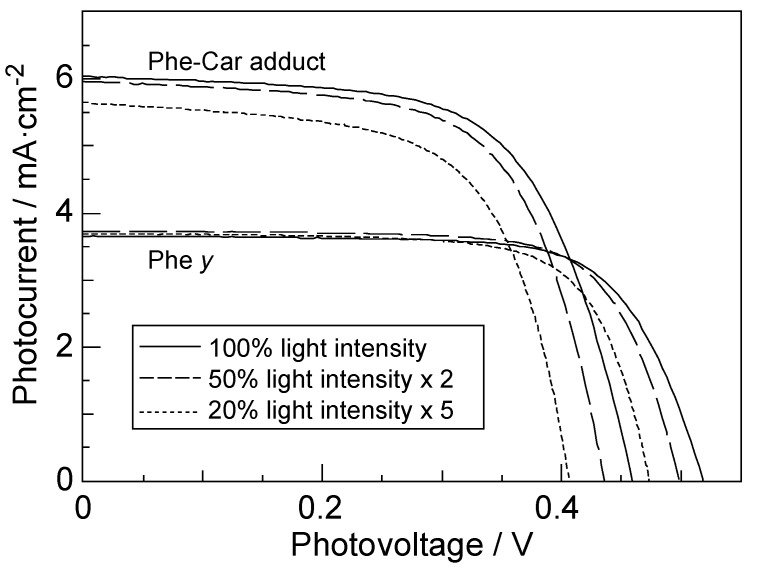
The light-intensity dependence of the *I−V* curves in solar cells using the Phe *y* and Phe–Car adduct sensitizers (reprinted from [9] with permission of Elsevier).

Figure 18 compares the light-intensity dependence of the *I*–*V* curves of solar cells using the Phe *y* and Phe–Car adduct sensitizers [9]. 

**Figure 19:**
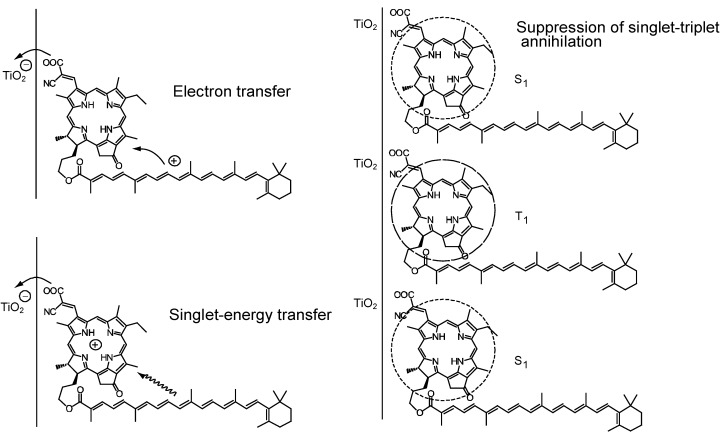
Mechanisms of the enhancement of photocurrent and conversion efficiency in the solar cell using the Phe–Car adduct sensitizer (reprinted from [9] with permission of Elsevier).

In the former, no clear changes in *J*_sc_ is seen even by lowering the light intensity into ^1^/_5_, whereas in the latter, systematic decrease in *J*_sc_ is seen as expected. The changes are somewhat comparable to the case of polyenes (see Figure 13): the light-intensity dependence of Phe *y* is similar to that of (MeO)_3_-*Φ*-6-CA (except for 100%), whereas that of Phe–Car adduct, to that of *Φ*-6-CA. The results indicate that some aggregation to cause singlet-triplet annihilation is formed in the Phe *y* sensitizer, whereas practically no aggregates are formed in the Phe–Car adduct sensitizer. Figure 19 pictorially proposes the mechanisms of enhancement in photocurrent/conversion efficiency on going from the Phe *y* to the Phe–Car adduct sensitizer, which include (i) electron transfer and (ii) singlet-energy transfer from the Car to the Phe *y* moiety as well as (iii) the suppression of the singlet-triplet annihilation reaction by preventing the aggregate formation by the use of the bulky Car moiety as a spacer.

Both singlet-energy transfer and electron transfer from the Car to the Phe moiety have been realized in the Phe–Car adduct. The photocurrent (*J*_sc_) was enhanced by 1.6 times, the photovoltage (*V*_oc_) was lowered by 0.9 times and, as a result, the conversion efficiency (*η*) was enhanced by 1.3 times. The *π*-conjugated chain of the Car moiety prevented the aggregate formation of the Phe moiety so that no sign of singlet-triplet annihilation was observed. Therefore, the Phe–Car adduct is potentially an excellent sensitizer to be used in a more refined way; the addition of short polyene spacers would improve the coverage of the TiO_2_ layer and to enhance the photovoltage (*V*_oc_), for example.

### 3.2. Chl c (Mg-Pheophorbide c) Sensitizers Having Porphyrin Skeleton

**Figure 20:**
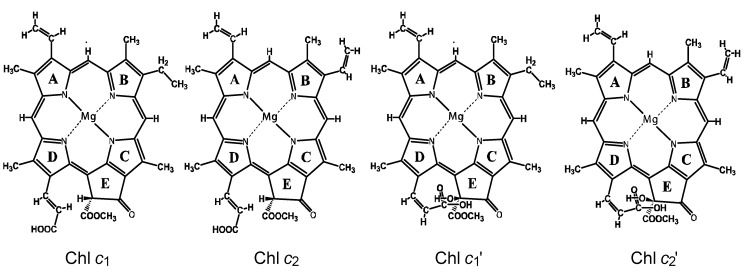
Chemical structures of Chl *c*_1_, Chl *c*_2_, Chl *c*_1_’ and Chl *c*_2_’ (reprinted from [7] with permission of MDPI Publishing).

Figure 20 presents the chemical structures of the pairs of Chls *c* and Chls *c*’ extracted from a sea weed called ‘*Undaria pinnatifida* (Wakame)’ [7]. The structures were determined by mass spectrometry and ^1^H-NMR spectroscopy. The latter includes rotating-frame Overhauser effect spectroscopy (ROESY) measurement to determine the nuclear Overhauser effect (NOE) correlations [10]. Chl *c*_1_ (Chl *c*_1_’) and Chl *c*_2_ (Chl *c*_2_’) have an ethyl group and a vinyl group, respectively, attached to ring B in different conformations. Further, Chl *c*_1_ and Chl *c*_2_ (Chl *c*_1_’ and Chl *c*_2_’) have hydrogen (hydroxyl group) attached to ring E, and also the carboxyl group attached to ring D through the vinyl group in the *cis* (*trans*) conformation with respect to a single bond attached to ring D. Thus, Chl *c*_1_’ and Chl *c*_2_’ can form intramolecular hydrogen bonding between the hydroxyl and carboxyl groups. Importantly, the chemical-shift values of the vinyl H suggest that the electron density is in the order, Chl *c*_2_ > Chl *c*_1_ > Chl *c*_2_’ > Chl *c*_1_’.

**Figure 21:**
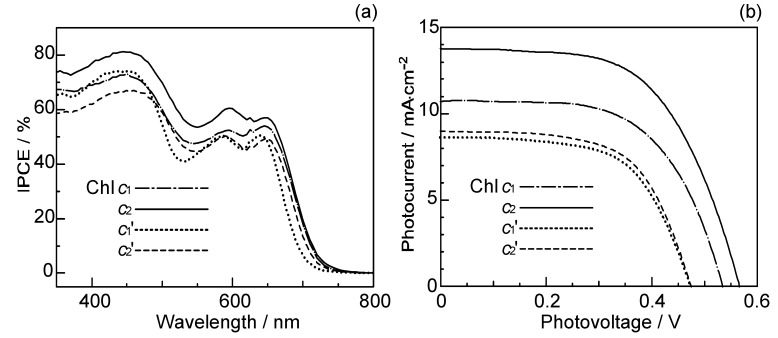
(**a**) The IPCE profiles and (**b**) the *I–V* curves of solar cells using the Chl *c*_1_, Chl *c*_2_, Chl *c*_1_´ and Chl *c*_2_´ sensitizers (reprinted from [10] with permission of Elsevier).

Figures 21a,b shows the IPCE profiles and the *I*–*V* curves, respectively, for solar cells using the set of four sensitizers [10]. The values of *J*_sc_ and *η* decrease, all in the order, Chl *c*_2_ > Chl *c*_1_ > Chl *c*_2_’ ≥ Chl *c*_1_’; the *V*_oc_ value also decreases in the same order. Interestingly, the decreasing order is in agreement with that of the electron density on the vinyl H, suggested by the H-chemical-shift values, but not necessarily with that of *the decreasingorder* of *E*_ox_, *i.e.*, Chl *c*_1_ > Chl *c*_2_ > Chl *c*_1_’ > Chl *c*_2_’.

**Figure 22:**
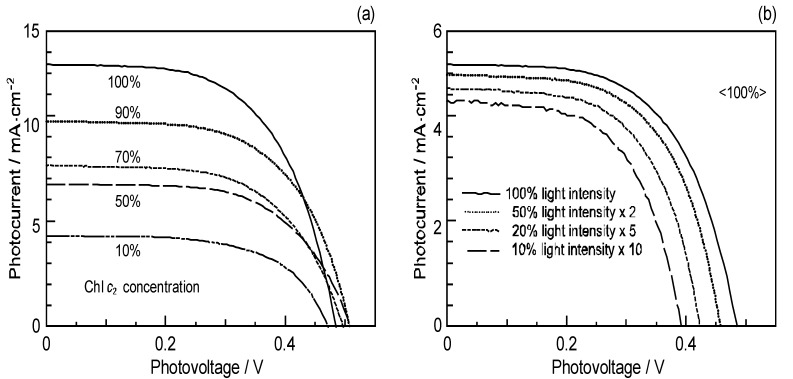
(**a**) Concentration dependence and (**b**) the light-intensity dependence of the *I–V* curves in Chl *c*_2_-sensitized solar cell (reprinted from [10] with permission of Elsevier).

Concerning the Chl *c*_2_-sensitizerd solar cell, Figure 22a shows that the photocurrent (*J*_sc_) and conversion efficiency (*η*) monotonously decreased toward the lower dye concentration, whereas Figure 22b shows that both the *J*_sc_ and *V*_oc_ values decreased toward the lower light intensity. There is no sign of singlet-triplet annihilation reaction due to the aggregate formation at all, in this particular sensitizer [10].

Chl *c*_2_ (Mg-Phe *c*_2_) has exhibited the highest photocurrent (*J*_sc_ = 13.8 mA∙cm^–2^) and conversion efficiency (*η* = 4.6%) among all the sensitizers we have tested. It is rather surprising that Phe *c*_2_ showed one of the lowest photocurrents (*J*_sc_ = 6.6 mA∙cm^–2^) and conversion efficiency (*η* = 1.1%), although their electronic absorption spectra are similar to each other. Most importantly, however, the one-electron oxidation potential of Chl *c*_2_ (1.06 V) is much lower than that of Phe *c*_2_ (1.33 eV).

## 4. Mechanism of Suppression and Enhancement of Photocurrent/Conversion Efficiency by Cosensitization of Pheophorbide Sensitizers without and with Metal, Mg or Zn

**Figure 23:**
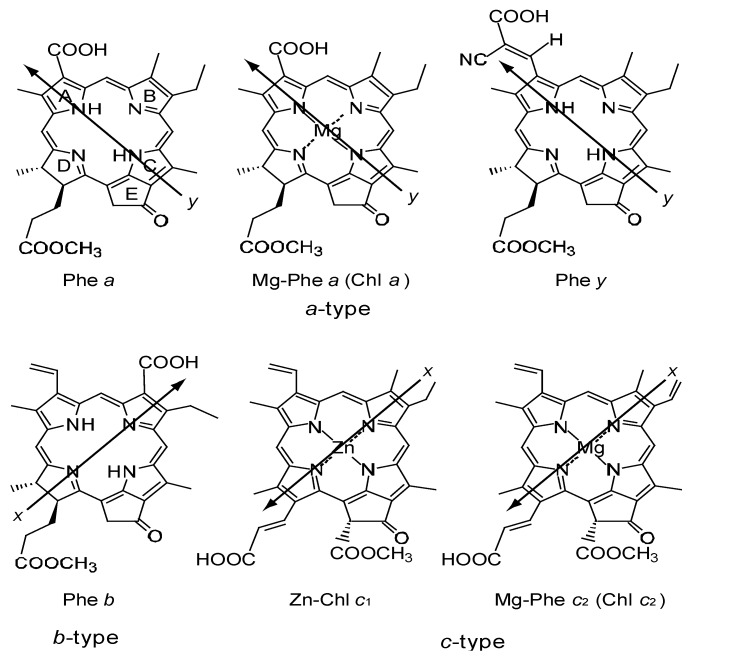
Chemical structures of Phe sensitizers without and with metal, Mg or Zn (reprinted from [7] with permission of MDPI Publishing).

Figure 23 presents the structures of sensitizers used in this section [7]. The structures can be characterized from two different viewpoints: *(a) The type of macrocycle.* The sensitizers can be classified into *three* different categories: (i) Phe *a*, Mg-Phe *a* (Chl *a*) and Phe *y*, having the chlorin macrocycle like Chl *a*, can be classified into the ‘*a*-type’ sensitizers. (ii) Phe *b* consisting of the chlorin macrocycle, to which a pair of C=O groups is. *(b) The positions of the carboxyl group*: The sensitizers can be classified into *three* different attached in the diagonal positions like Chl *b* (Mg-Phe *b*), can be classified into the ‘*b*-type’ sensitizer. (iii) Zn-Phe *c*_1_ and Mg-Phe *c*_2_ (Chl *c*_2_) having the porphyrin macrocycle like Chl *c*, can be classified into the ‘*c*-type’ sensitizersgroups in terms of the positions of the carboxyl group. (i) The carboxyl group is directly attached to ring A in Phe *a* and Mg-Phe *a*, but through an additional double bond in Phe *y*, (ii) it is attached to ring B in Phe *b*, and (iii) it is attached to ring D through a double bond in Zn-Phe *c*_1_ and Mg-Phe *c*_2_ (Chl *c*_2_). In terms of the *x*-axis and the *y*-axis that have been originally defined for the Q*_x_* and Q*_y_* transitions of Chl *a*, the carboxyl group is on the *y*-axis in Phe *a*, Mg-Phe *a* and Phe *y*, whereas it is on the *x*-axis in Phe *b*, Zn-Phe *c_1_* and Mg-Phe *c_2_*. See the sticks of arrows directing to the carboxyl group, which can be classified into two directions.

**Figure 24:**
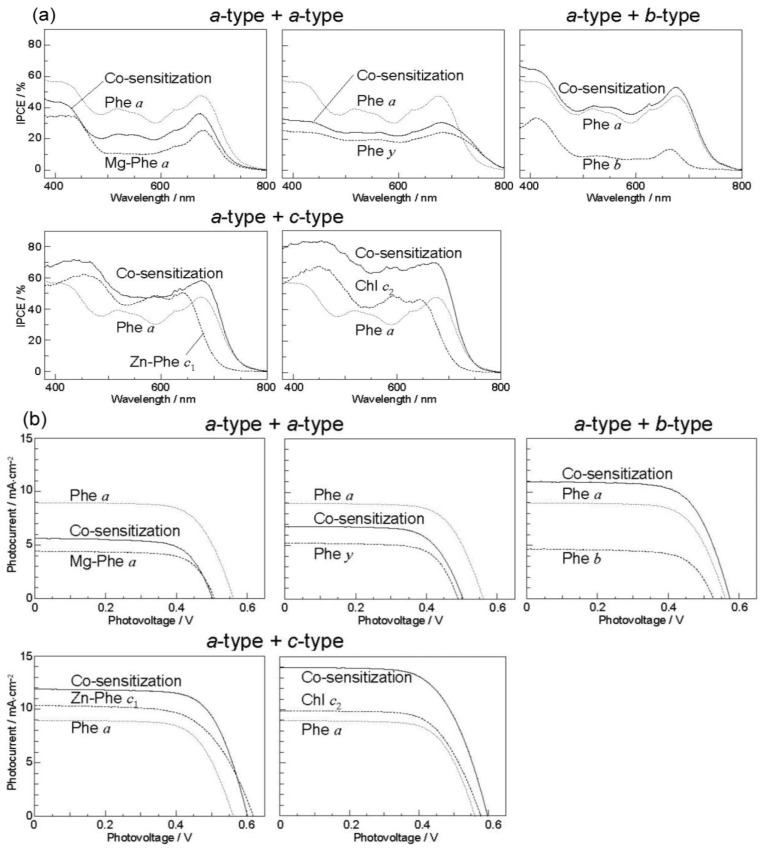
(**a**) The IPCE profiles for three different types of co-sensitization and (**b**) the *I–V* curves for three different types of co-sensitization.

Figure 24 exhibits (a) the IPCE profiles and (b) the *I*–*V* curves for the five pairs of sensitizers, which can be classified into *three* different types of cosensitization, *i.e.*, *a*-type + *a*-type, *a*-type + *b*-type and *a*-type + *c*-type. 

In the present experiments of cosensitization, Phe *a* was used as the principal sensitizer in common. The IPCE profiles and the *I*–*V* curves *pictorially* demonstrate that the cosensitization (shown in solid line) of *a*-type + *a*-type gives rise to the suppression, whereas those of *a*-type + *b*-type and *a*-type + *c*-type give rise to the enhancement of photocurrent/conversion efficiency.

Table 4 lists the *V*_oc_, *FF*, *J*_sc_ and *η* values for the *singly-sensitized* solar cells and the *E*_ox_ values for the principal and individual cosensitizers (see the captions for their definitions of abbreviated parameters). In comparison to the principal sensitizer, the *J*_sc_ and *η* values are smaller in *a*-type and *b*-type cosensitizers, but they are larger in *c*-type cosensitizer. The *V*_oc_ and *FF* values are more or less similar among the set of sensitizers.

**Table 4:** The open-circuit photovoltage (*V*_oc_), fill factor (*FF*), short-circuit photo-current density (*J*_sc_), conversion efficiency (*η*) of the singly-sensitized solar cells and the one-electron oxidation potential (*E*_ox_) of each sensitizer.

The standard and co-sensitizer	*V_oc_* / V	*FF*	*J_sc_* / mA·cm^–2^	*η*	*E_ox_* / V *vs*. NHE
Phe *a* (*a*-*type*)	0.56	0.68	9.0	3.4	1.16
*a-type*					
Mg-Phe *a*	0.51	0.70	4.4	1.6	0.79
Phe *y*	0.49	0.70	5.2	1.8	1.19
*b-type*					
Phe *b*	0.53	0.70	4.6	1.7	1.24
*c-type*					
Zn-Phe *c*_1_	0.62	0.63	10.4	4.0	1.16
Mg-Phe *c*_2_ (Chl *c*_2_)	0.58	0.66	13.8	4.6	1.06

Table 5 lists the *V*_oc_, *FF*, *J*_sc_ and *η* values for the cosensitized solar cells using *a*-type, *b*-type and *c*-type as the cosensitizers. Cosensitization of the principal sensitizer with *a*-type sensitizers give rise to lower *J*_sc_ and *η* values, whereas cosensitization with *b*-type and *c*-type sensitizers, give rise to definitively higher *J*_sc_ and *η* values. The enhancement factors ^r^*J*_sc_ and ^r^*η* (defined below the table) are definitely higher (lower) in the latter (former) cosensitizations. Spectral separation, *S*, is also listed in the last column with its definition at the bottom.

**Table 5:** The open-circuit photovoltage (*V*_oc_), fill factor (*FF*), short-circuit photo-current density (*J*_sc_), conversion efficiency (*η*) of the solar cells co-sensitized, and the spectral separation (S). The definitions of ^r^*J*_sc_ and ^r^*η* are given below the table.

Co-sensitizers	*V_oc_* / V	*FF*	*J*_sc_* /* mA·cm^–2^	^r^ *J*_sc_	*η*	^r^ *η*	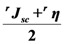	*S*
*a-type*								
Mg-Phe *a*	0.50	0.69	5.6	0.83	1.9	0.76	0.8	41
Phe *y*	0.50	0.68	6.8	0.97	2.3	0.88	0.9	62
*b-type*								
Phe *b*	0.57	0.68	10.9	1.60	4.3	1.65	1.6	39
*c-type*								
Zn-Phe *c*_1_	0.60	0.69	11.9	1.23	5.0	1.35	1.3	80
Mg-Phe *c*_2_ (Chl *c*_2_)	0.60	0.64	14.0	1.47	5.4	1.50	1.5	95

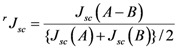
; 
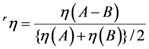

Concerning cosensitization, the *three* different pairs of sensitizers give rise to suppression or enhancement in reference to the average of performance of the component sensitizers: (i) The *a*-type + *a*-type cosensitization gives rise to suppression of performance; the relative performance values decrease for both sensitizers, *i.e.*, Mg-Phe *a* (*^r^J*_sc_ = 0.83, *^r^η* = 0.76) and Phe *y* (*^r^J*_sc_ = 0.97, *^r^η* = 0.88), the averaged ratios being ~0.8 and ~0.9, respectively. (ii) The *a*-type + *b*-type cosensitization with the cosensitizer, Phe *b*, shows remarkably-high enhancement (*^r^J*_sc_ = 1.60, *^r^η* = 1.65), the averaged ratio being 1.6. (iii) The *a*-type + *c*-type cosensitization causes large enhancement with the sensitizers, Zn-Phe *c*_1_ (*^r^J*_sc_ = 1.23, *^r^η* = 1.35) and Mg-Phe *c*_2_ (*^r^J*_sc_ = 1.47, *^r^η* = 1.50), the averaged ratio being ~1.3 and ~1.5, respectively. Importantly, the combination of the chlorin (Phe *a*) and the porphyrin (Mg-Phe *c*_2_) sensitizers, each showing the highest two individual performance, gave rise to the highest enhancement of the *J*_sc_ value (9.0 and 13.8 → 14.0 mA·cm^–2^) and the *η* value (3.4 and 4.6 → 5.4%).

**Figure 25:**
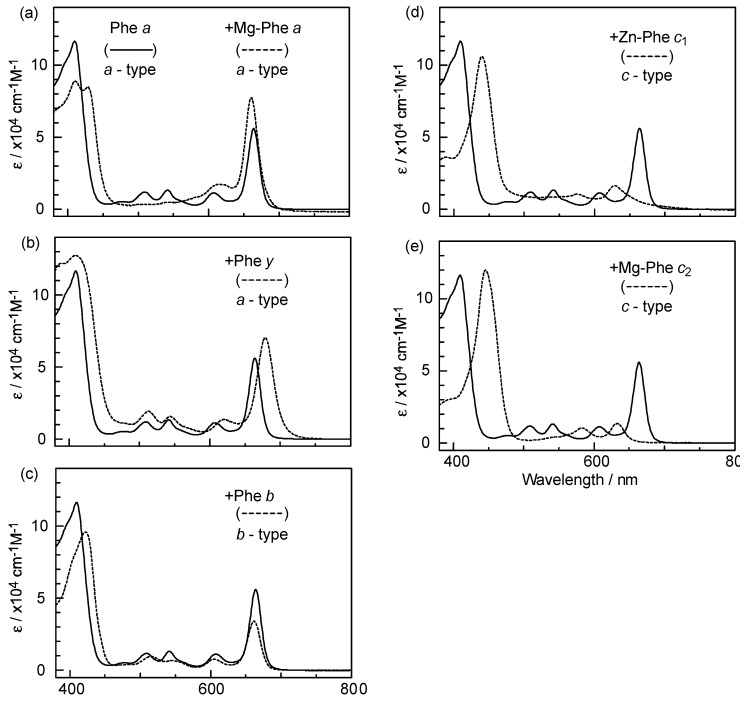
The electronic absorption spectra of the pairs of co-sensitizers in ethanol solution. (**a**) Phe *a* + Mg-Phe *a*; (**b**) Phe *a* + Phe *y*; (**c**) Phe *a* + Phe *b*; (**d**) Phe *a* + Zn-Phe *c*_1_ and (**e**) Phe *a* + Mg-Phe *c*_2_ (reproduced with permission from [7] of MDPI Publishing).

Figure 25 shows the electronic-absorption spectra of the pairs of sensitizers in ethanol solution [7], which can be characterized as follows: *Individual sensitizers*: Chlorin sensitizers of both *a*-type (Mg-Phe *a* and Phe *y*) and *b*-type (Phe *b*) clearly exhibit the Soret, Q*_x_* and Q*_y_* absorption peaks, whereas the metal-porphyrin sensitizers of *c*-type (Zn-Phe *c*_1_ & Mg-Phe *c*_2_) exhibit the Soret peak on the longer-wavelength side and a pair of peaks (possibly Q*_x_* and Q*_y_*) on the shorter-wavelength side. *A pair of cosensitizers*: Depending on the overlapped and split absorption peaks due to the pair of sensitizers, competitive or complementary light absorption is expected to take place. Concerning the overlap of cosensitizer absorption peaks, (i) the ‘*a*-type + *a*-type’ cosensitizer pair and the ‘*a*-type + *b*-type’ pair are overlapped in a complicated ways. However, (ii) the ‘*a*-type + *c*-type’ pair exhibits no overlaps in either the Soret or the Q*_y_* absorptions. To evaluate the overlap over the spectral region, we have defined ‘spectral separation (*S*)’:
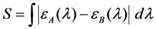
(1) and the values are listed in Table 5 (right end). Importantly, it is rather small in the *a*-type + *a*-type and *a*-type + *b*-type pairs and the largest in the *a*-type + *c*-type pairs.

**Figure 26:**
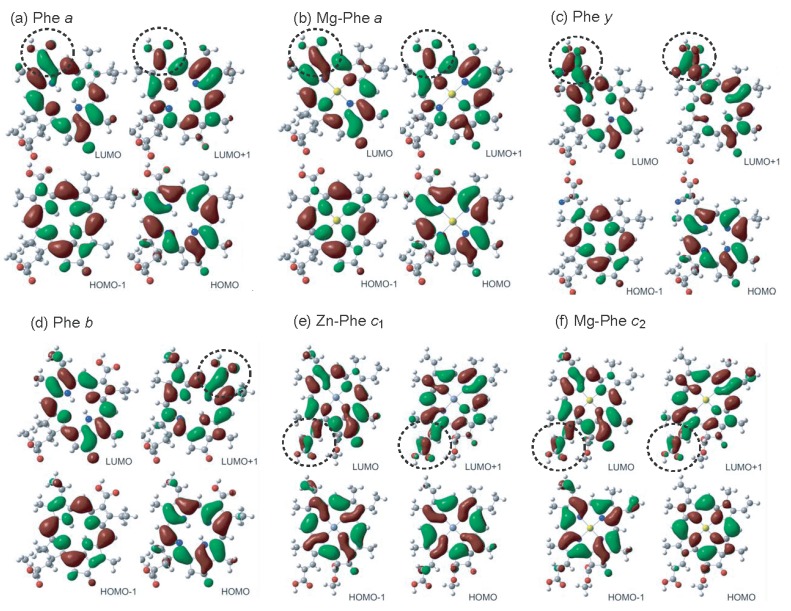
The four orbitals including HOMO–1, HOMO, LUMO and LUMO+1 obtained by TD-DFT calculations (reproduced from [7] with permission of MDPI Publishing).

We examined the effects due to the type of macrocycles and the position of the carboxyl group on the molecular orbitals by means of the time-dependent density-function-theory (TD-DFT) calculations: Figure 26 shows the calculated four major molecular orbitals, including HOMO–1, HOMO, LUMO and LUMO+1 (here, HOMO and LUMO stands for the highest-occupied molecular orbital and the lowest-unoccupied molecular orbital, respectively). 

The shapes of the four molecular orbitals are different depending on the type of macrocycle, chlorin or porphyrin. The LUMO and LUMO+1, that are expected to play the key role in the electron injection into TiO_2_, are found to be extended toward the carboxyl group; in other words, the electron density is shifted toward the carboxyl group to get ready for electron injection (see the regions shown in dotted circles). Also, the electronic transitions are mainly determined by the combination of the {HOMO–1, HOMO} → {LUMO, LUMO+1} transitions and, therefore, all the Soret, Q*_x_* and Q*_y_* transitions are expected to be strongly influenced by the position of the carboxyl group (or, in other words, by the direction of polarization).

The results of DFT calculations shown in Figure 26 [7] have provided us with a strong support to the ideas that the type of macrocycle, chlorin or porphyrin, and the position of the carboxyl group, on the *y*-axis or the *x*-axis, strongly affect the directions of electron-injection and transition-dipole moment.

**Figure 27:**
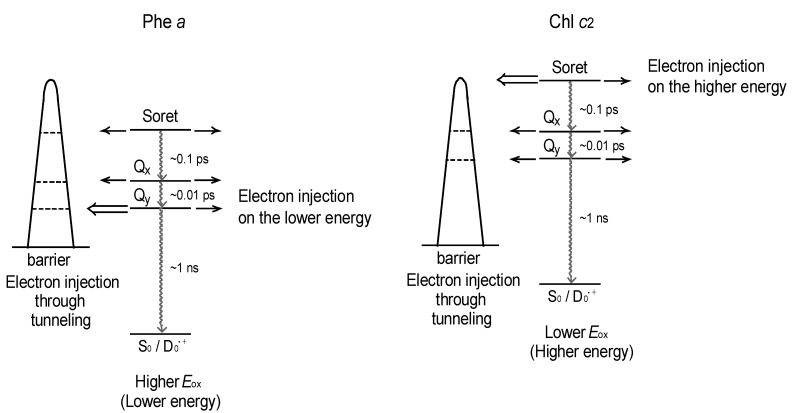
Effects of the one-electron oxidation potential and the pattern on the electron-injection channels through the tunneling mechanism. No strong correlation between the electron injections from the Phe *a* and Chl *c*_2_ sensitizers are expected (reproduced from [7] with permission of MDPI Publishing).

The suppression or enhancement of performance in cosensitization can be explained in terms of the light absorption (competitive or complementary), the direction of transition-dipole moment (parallel or orthogonal) and the singlet-energy transfer (interactive or independent) between the pair of sensitizers:

(i) The absorption spectra of the sensitizers (in Figure 25) show that the major light absorptions are *absolutely* complementary in the *a*-type + *c*-type pair. Therefore, the highest enhancement in the *a*-type + *c*-type cosensitization can be rationalized in terms of complementary absorption *not only* in the Q*_x_* and Q*_y_* levels *but also* in the Soret levels.
(ii) The combination of the *a*-type sensitizer having the carboxyl group in the *y*-direction and the *b*-type or *c*-type sensitizer having the carboxyl group in the *x*-direction can give rise to the highest enhancement of photocurrent and conversion efficiency, because of the minimum interference of the transition dipoles between the pair of cosensitizers. Polarization and electron-injection along the orthogonal directions must prevent the interference between the intermolecular transition dipole–transition dipole interactions that can trigger intermolecular energy transfer and the resultant dissipation of the singlet energy.
(iii) A pair of electron injections through energetically different levels,*i.e.*, one, the lower level of the Q*_y_* absorption of Phe *a* and the other, the higher level of the Soret absorption of Chl *c*_2_ (Mg-Phe *c*_2_) is expected to have little interference between each other (see Figure 27 [7]).

Cosensitization by the use of the best and the second-best sensitizers, *i.e.*, Chl *c*_2_ (Mg-Phe *c*_2_) and Phe *a*, we have achieved the maximum enhancement in photocurrent (*J*_sc_ = 14.0 mA·cm^–2^) and conversion efficiency (*η* = 5.4%), the enhancement factor being 1.47 and 1.50 times in reference to the averaged value of the performance of the component cosensitizers. The enhancement is ascribed to the complementary light absorption, the orthogonal transition-dipole moments and the energetically different pathways of electron injection.

## 5. Regression: Singlet-Triplet Annihilation Reaction

We have provided specialized readers with enough information, extracted from the raw data, concerning the mechanisms of suppression and enhancement of photocurrent/conversion efficiency (performance) in dye-sensitized solar cells using carotenoid and chlorophyll derivatives as sensitizers (the title of this review). However, it might be worthwhile, for non-specialized readers, to add ‘a regression section’ concerning one of the most important subjects in this review, *i.e.*, ‘singlet-triplet annihilation’, by referring to and correlating Figures and Tables according to the order of the previous sections and subsections, after an easy Introduction. Those readers who are interested are encouraged to read the previous Section 1,Section 2,Section 3 again according to our guide.

### 5.1. An Easy Introduction to Mechanisms of Singlet-Triplet Annihilation

**Figure 28:**
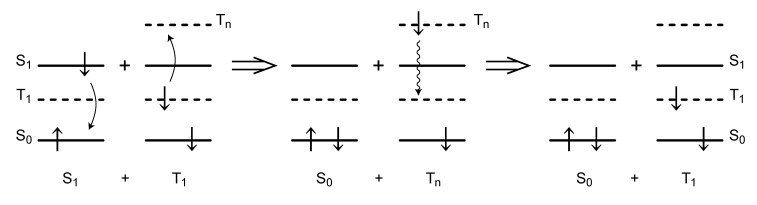
Mechanism of singlet-triplet annihilation. When a pair of the S_1_ and T_1_ states becomes close-by, a resonance electron transfer takes place, one downward and the other upward. As a result, the S_1_ singlet-excited state becomes annihilated and the T_1_ state becomes excited to an upper T*_n_* triplet state (the left → middle transformation; an overall S_1_ + T*_n_* state is generated). Then, the T*_n_* state quickly relaxes down to the T_1_ state, because the inversion of spin is unnecessary (the middle → right transformation; an overall S_0_ + T_1_ state resulted). Through this two-steps of reaction, the S_1_ → S_0_ singlet transformation is mediated by the T_1_ state, which is called ‘singlet-triplet annihilation’.

There are two different kinds of electronic states, singlet and triplet, with anti-parallel and parallel pairs of spins. The names originate from their number of splittings, when placed under the external magnetic field. The most stable state is the ground state (S_0_), where a pair of anti-parallel spins is present on the same lowest electronic level. When one of the spins goes to the next upper state, it becomes the singlet excited-state (S_1_). When the inversion of the upper spin takes place to form a parallel spin, it becomes the lower triplet state (T_1_). Then, the overall state is S_1_ + T_1_, from which the ‘singlet-triplet annihilation’ reaction starts, as shown in Figure 28.

When the S_1_ and T_1_ states become close-by, a resonance electron transfer takes place, one downward and the other upward. As a result, the singlet-excited state (S_1_) is annihilated and the T_1_ state becomes excited to an upper state (T*_n_*) (as shown in the left end of the figure). Then, the T*_n_* state can quickly relaxes down to the T_1_ state, because the inversion of spin is unnecessary (as shown in the middle). As an overall reaction, the S_1_ excitation, which can inject the upper electron to a semiconductor TiO_2_ in our solar cells, becomes annihilated (as shown in the right-end). The resultant T_1_ state is now ready to annihilate a next S_1_ excitation.

The most important difference between the S_1_ and T_1_ states is their lifetimes. The S_1_ state can relax down to the S_0_ state instantaneously because no inversion of spin is necessary, whereas the T_1_ state takes ~10^4^ longer time to relax down to the S_0_ state, because the inversion of the upper spin is necessary under the influence of the external magnetic field.

This ‘single-triplet annihilation’ reaction can take place, only when the pair of the S_1_ and T_1_ excitations becomes close-by. When the interaction between them is *not strong enough* or *sterically hindered*, the singlet-triplet annihilation reaction can never take place.

### 5.2. A Brief Summary of Section 1,Section 2,Section 3,Section 4, Focusing on the Singlet-Triplet Annihilation Reaction

In Section 1, the conjugation-length dependence in photocurrent/conversion efficiency (performance) of DSSCs, using RA and CAs sensitizers (Figure 1), was determined by the use of *I*–*V* curves (Figure 2). The performance was the highest in CA7, and declined toward both RA5 and CA13 (Figures 3a,b). The latter decrease was nicely explained in terms of the pathways and time constants of electron injection immediately after excitation, *i.e.*, <10 ps, for CA7–CA11 (Figure 5). The electron-injection efficiencies are listed in Table 1 and presented in Figure 3c. However, the decline toward RA5 (Figure 3a,b) was left to be explained.

This decline toward the shorter chain was explained by analyzing the later stages after excitation, *i.e.*, <40 μs, as presented in Figure 6 and Table 3. It was found that a combined D_0_^●^^+^ + T_1_ state split into a mixture of the D_0_^●^^+^ state and T_1_ state (Figure 6). Here, the T_1_/D_0_^●^^+^ ratio was similar between the combined state and the split states, which indicates that the T_1_/D_0_^●^^+^ ratio is determined by the energy gap between the conduction band edge (CBE) and the T_1_ state shown in Figure 7.

It is important that the yield of the D_0_^●^^+^ state generated by electron injection decreased, whereas that of the T_1_ state increased systematically in the order, CA8 → CA7 → CA6 → RA5 bound to TiO_2_ (Table 3).

In Section 2, we tried to obtain more direct evidence for the presence of singlet-triplet annihilation: In Subsection 2.1, the dependence of performance on the dye concentration in CA7-sensitized solar cell is presented. A unique concentration dependence is seen in the *I*–*V* curves shown in Figure 9. The photocurrent, *J*_sc_, and the conversion efficiency, *η*, are plotted as the functions of the CA7 concentration in Figure 10. The highest performance was seen at 70% and the second-highest, at 30%; both were higher than the value at 100%.

In Figure 10, key changes were characterized in terms of (i) coherent, completely delocalized excitation at 100%, (ii) partially-destroyed delocalized excitation at 90%, (iii) localized migrating excitation at 70%, and (iv) a maximum isolated excitation at 30%. The typical arrangement of the dye (○) and the spacer (●) molecules depicted in Figure 11 was used for this explanation.

In Subsection 2.2, much more convincing evidence for the singlet-triplet annihilation reaction was obtained by the use of a set of medium-sized conjugated CAs having different transition-dipole moments (Figure 12). (a) The concentration dependence and (b) the light-intensity dependence of the photocurrent (presented in Figure 13) and the conversion efficiency (Figure 14) showed the following trends in the least-polarizable dye sensitizer, *i.e.*, *Φ*-6-CA. Both the photocurrent and conversion efficiency decreased toward the lower dye concentration or the lower light intensity, as we expect.

On the other hand, both the photocurrent and the conversion efficiency increased toward the lower dye concentration and the lower light intensity, in the most-polarizable dye sensitizer, *i.e.*, Me_2_N-*Φ*-CA, contrary to our expectation. Concerning the latter, the two different concentrations of the dye sensitizer were used. This observation can be explained only in terms of ‘singlet-triplet annihilation reaction’ that is most efficient at the higher dye concentration or at the higher light intensity.

In Section 3, the first type of dependence, as we expect, can be used to show the absence of singlet-triplet annihilation. In Subsection 3.1, it was shown that no singlet-triplet annihilation took place (as shown in Figure 18) due to the steric hindrance of the straight CA sidechain of the Phe–Car adduct sensitizer (Figure 19). In Subsection 3.2, no sign of singlet-triplet annihilation took place, either (Figure 22), due to the rigid and flat porphyrin macrocycle in the Chl *c*_2_ sensitizer (Figure 20).

Thus, the absence (or presence) of the dependence on the dye concentration or the light intensity, contrary to our expectation, can be used to determine the absence (or presence) of the singlet-triplet annihilation reaction.
